# Does COVID-19 Promote Self-Service Usage among Modern Shoppers? An Exploration of Pandemic-Driven Behavioural Changes in Self-Collection Users

**DOI:** 10.3390/ijerph18168574

**Published:** 2021-08-13

**Authors:** Xueqin Wang, Yiik Diew Wong, Kum Fai Yuen

**Affiliations:** 1 Department of International Logistics, Chung-Ang University, Seoul 06974, Korea; Xueqinwang@cau.ac.kr; 2 School of Civil and Environmental Engineering, Nanyang Technological University, Singapore 639798, Singapore; cydwong@ntu.edu.sg

**Keywords:** self-service, last-mile logistics, pandemic-driven behavioural change, self-collection locker, e-commerce delivery, COVID-19

## Abstract

Due to health concerns related to COVID-19, shoppers have learned to minimise social contact by adopting various contactless self-service technologies to fulfil their consumption needs. This study explores shoppers’ behavioural changes in relation to self-service, using the special research context of e-commerce self-collection services. By synthesising insights from the health psychology literature, this study proposes an affective-cognitive-social perspective to explain the pandemic-driven behavioural changes of self-collection users. The survey instrument is used for online data collection (*n* = 500), and a combined (descriptive and quantitative) method is adopted for data analysis. Our results suggest that, although with a relatively weak predictive power, the affective and cognitive appraisals of health risks lead to the reinforced usage of self-collection service. This also applies to the factors of action/coping planning and subjective norm. This study theoretically contributes to the self-service literature and creates managerial implications for retailers and logistics operators.

## 1. Introduction

The COVID-19 pandemic has been a major disruption that has changed shoppers’ behaviours [1,2]. Due to pandemic-induced health concerns, shoppers have learned to minimise social contact by adopting various contactless shopping/delivery technologies to fulfil their consumption needs [3]. These technologies may include the following: virtual voice assistants or chatbots that guide shoppers’ information search before shopping [4,5]; e-commerce or mobile-commerce platforms along with self-checkout portals for purchasing and, more pertinent to the current study, unmanned self-collection facilities for parcel deliveries after shopping [6,7]. As acutely observed by some researchers, the pandemic has catalysed a global trend of digitalisation at an unprecedented rate, leading to a shift from a high-touch to a high-tech orientation in the service industry [8,9]. 

Apart from an apparent heightened reliance on technologies among shoppers, a parallel but less explicit trend characterised by *self-dependence* or *self-service* seems to exist. Such a trend may be partly due to the need to comply with social distancing practices which are gradually routinised into shoppers’ daily activities (e.g., preference of self-collect deliveries to avoid unnecessary contact with delivery personnel). It may also be due to the service disruptions/failures caused by the pandemic that enhance shoppers’ need for taking control (i.e., preference of self-collect deliveries if home deliveries are likely to be disrupted/delayed). Furthermore, the self-serve spirit is also essential to the individualistic culture emerging within modern society [10,11,12]. Even before the pandemic, modern shoppers had been found to lead an increasingly private life while interacting with each other more actively in the virtual world. Moreover, the pandemic, along with various distancing measures, has further accustomed modern society to such an individualised lifestyle [13,14,15]. Indeed, a recent review by Wang, Wong and Yuen [2] suggests a rising segment of ‘lonely’ consumers (without a negative connotation) along with the pandemic; These consumers engage in consumption activities independently by using technological resources rather than relying on social supports. 

Therefore, the pandemic brings new perspectives into shoppers’ responses to self-service technologies (SSTs), where engaging with SSTs is more than a service consideration but of health and psychological importance [16,17]. To this end, a great amount of research has been directed towards addressing the critical role of ‘technologies’ in facilitating consumption activities under the context of social distancing [3,9,18]. Yet, the parallel element of ‘self-service’ has been left largely unnoticed. Although the expedited uptake of shopping technologies has almost been an observed fact during the pandemic [19,20,21], the question ‘Does COVID-19 promote a self-service spirit (via technologies) among modern shoppers?’ remains answered. Hence, our study answers this question by exploring shoppers’ behavioural changes concerning their engagement with SSTs, focussing on the pandemic-induced importance of self-service. 

The field of last-mile logistics delivery represents a fitting context to the current study [22,23]. To illustrate, conventional last-mile delivery has been dominated by direct home deliveries (i.e., shoppers being served by logistics operators). The introduction of self-collection services (i.e., shoppers self-collecting deliveries from nearby facilities) faced great resistance from end users before the pandemic [24]. To use self-collection services, shoppers are expected to invest additional time and effort in visiting the collection facilities, retrieving parcels from the password-protected lockers and transporting the parcels back home. In return, shoppers take control of the time and location of parcel collection rather than wait at home for hours to attend to home deliveries. Notably, self-collection services incorporate the now-commonplace locker technique, which is low in technological complexity to modern shoppers. Thus, the lack of self-service motivation (vs. technological complexity) may be the primary obstacle discouraging shoppers from using the service, which is the key emphasis of our study. Indeed, last-mile logistics has always been viewed as a business-dominant field where shoppers are used to being *served*, and eliciting shoppers’ participation through self-service has been challenging [25,26]. Hence, it would be worthwhile to attempt to explore whether the pandemic has promoted the shoppers’ self-service spirit in last-mile logistics, thus driving the uptake of self-collection services. 

On the basis of a theoretical premise, this study synthesises insights from the health literature to explain shoppers’ behavioural changes in relation to self-service [27,28,29,30,31,32,33,34]. Firstly, the health literature posits risk appraisals as a critical factor leading to individuals’ behavioural changes [28,29,30]. In this connection, we adopt Sheeran, Harris and Epton’s [29] dual conceptualisation, which categorises risk appraisals into an affective dimension (i.e., anticipatory emotion and anticipated emotion) and a cognitive dimension (i.e., perceived severity and perceived susceptibility). Secondly, planning is another critical cognitive factor when implementing behavioural changes [31,32]. In line with this stream of studies, we explore the roles of action planning and coping planning in shoppers’ usage of self-collection services. Finally, the social impact of the subjective norm is also considered in changing shoppers’ self-service patterns [33,34]. Therefore, with the synthesised effort, our study contributes to the literature with an affective–cognitive–social perspective of self-service usage among modern shoppers. By doing so, this study enriches the self-service literature with insights from the health psychology/behaviour literature, which has become increasingly salient but seldom been addressed in the context of shopping services. At the same time, we also contribute to the literature by extending the theoretical premises originated from health studies to the commercial service context. This is achieved by validating and comparing the predictive power of factors concerning health behaviour (e.g., risk appraisals, planning and social influence) in a selective context of self-service.

The remainder of this manuscript is structured as follows. Section 2 provides the review of related literature and rationalises the self-collection services as a fitting research context to the current study. Several hypotheses are also proposed by synthesising insights from the health literature. Next, Section 3 elaborates on the research method, including the questionnaire design and the survey administration process. Section 4 then presents the research findings. Finally, Section 5 discusses this study’s theoretical and managerial implications. 

## 2. Literature Review

Extant studies on the self-collection service are examined. We highlight the service’s compatibility with the pandemic situation and rationalise it as an ideal research context to examine pandemic-driven self-service among shoppers. Moreover, various streams of health literature are synthesised to explain shoppers’ behavioural change patterns in response to the pandemic. The affective, cognitive and social aspects are emphasised, and relevant hypotheses are developed. 

### 2.1. Research Context: Self-Collection Service

Self-service, and self-collection in particular, is not a new topic in the e-commerce or logistics literature. The self-collection service has been examined under different terms, including unmanned collection and delivery point [24,35,36], unattended pickup facility [37], automated parcel locker/station [38,39] and smart locker [6,7]. Although the specific terms may vary, they all refer to the same service that requires shoppers to visit collection facilities, retrieve parcels from lockers and carry the parcels home (or to the next destination) thereafter. 

Wang, Yuen, Wong and Teo [23] and Yuen, Wang, Ng and Wong [22] are among the first theoretical studies that examine shoppers’ adoption of self-collection services. From an innovation diffusion perspective, the perceived characteristics of self-collection services, such as the relative advantage, compatibility and complexity, predict shoppers’ adoption behaviour [22,23]. The pilot works were extended by recent studies incorporating various theories to explain consumers’ acceptance/resistance towards self-collection services. These include insights from theory of planned behaviour [7,40], resource matching [6,7], perceived value and transactional cost [6,26], service convenience [41,42], and technology anxiety and readiness [42]. As such, a multi-theory perspective of shoppers’ usage of self-collection services has been gradually established. 

Despite the rich insights generated, most existing research viewed the self-collection service as an innovative technology, where technological elements were emphasised as the motivators or obstacles for shoppers’ initial adoption. However, with years of commercialisation, the service may well have passed the initial stage of technology acceptance. After all, it employs a simple tool of parcel locker that is a commonplace technology to most modern shoppers. Thus, now is a good time to shift the research focus from the technological elements to the self-service elements, which are more salient considerations in shoppers’ usage of self-collection services. 

Furthermore, the service was initially introduced to address the ‘not-at-home’ issue in last-mile delivery and simultaneously enhance flexibility in parcel reception for end-consumers [43,44]. The pandemic; however, brings new benefits to the self-collection service. The locker-based collection service eliminates direct social interactions necessary for conventional home deliveries, leading to contactless and self-served collection experiences [3]. Consequently, the self-collection service becomes especially compatible with the pandemic situation of social distancing. To this end, the service serves as an ideal research context for examining pandemic-driven behavioural changes concerning self-service. 

Notably, a small stream of research has started to investigate shoppers’ adoption of contactless deliveries in response to the pandemic, such as delivery robots [45,46] and delivery apps [47,48]. Some researchers have even raised the following question: *Is the COVID-19 pandemic strong enough to change online order delivery methods?* [49]. However, these studies again focus primarily on the technological elements of contactless deliveries, applying theories such as innovation diffusion and technology acceptance [49]. To the best of the authors’ knowledge, no research has used the unique context of self-collection (i.e., low in technological complexity and high in self-service commitment) to examine the impacts of the pandemic on shoppers’ changing behaviours with respect to self-service. 

### 2.2. Theoretical Premise and Hypothesis Development 

The health literature offers rich insights into individuals’ behavioural changes in response to external hazards. Before establishing our theoretical premise, we must first define the patterns of behavioural changes addressed in this study. In this regard, we adapt the behaviour change support system of Oinas-Kukkonen [50]. To illustrate, in Oinas-Kukkonen’s [50] original work, three behavioural change patterns are identified, that is, the newly formed (i.e., non-users becoming users), the altered (i.e., increased or decreased behavioural intensity/frequency), and the reinforced (i.e., strengthened behaviour with/without marginal changes). To apply Oinas-Kukkonen’s [50] work to the current research context, several adjustments are necessary. In particular, the self-collection service has been introduced in the research region for more than five years. Considering the maturity level of the selective self-service context in the current study, we foresee a relatively small shopper group that corresponds to the newly formed category, which may not be adequately represented to produce meaningful comparisons. Thus, the ‘newly formed’ behavioural change pattern is removed from our study. Furthermore, to distinguish the shopper groups with increased and decreased usage frequencies of self-service, we split the original altered category into two sub-categories, which are labelled as the altered (with decreased frequency) and the reinforced (with increased frequency). In addition, the original ‘reinforced’ category is replaced with a more intuitive label of ‘the maintained’. 

As such, we propose that the pandemic leads to the following three behavioural change patterns based on usage frequencies of self-service: (a) the *reinforced* (i.e., usage frequency increased in response to the pandemic), (b) the *maintained* (i.e., usage frequency unchanged), and the *altered* (i.e., usage frequency decreased in response to the pandemic). Thus, drawing on theoretical insights from health research, our study aims to profile the self-collection users who demonstrate these three distinctive patterns of behavioural changes. 

#### 2.2.1. Risk Appraisals: Cognitive and Affective Components 

The first stream of health literature focuses on individuals’ risk appraisals. Several theories consider risk appraisals to be the key determinants to individuals’ behavioural changes [29]. For example, the health belief model (HBM) suggests that individuals’ assessments on their *susceptibility* to the health crisis and the associated *severity* of the consequence lead to health-related behaviours [51,52,53]. Here, the susceptibility and severity perceptions are the two constructs of risk appraisals, the former referring to the likelihood of being adversely affected and the latter concerning the seriousness of the outcome. The perceived susceptibility and severity are also key constructs in the protection motivation theory (PMT) and the extended parallel process model (EPPM) [54,55,56,57]. In line with HBM, both PMT and EPPM emphasise individuals’ cognitive appraisals on their vulnerability to the health risks and the severity of the health consequences, which motivate self-protective behaviours [14]. In the context of self-collection services, the perceived susceptibility and severity of contracting the COVID-19 virus may discourage shoppers from any direct social interactions. As a result, they may be more motivated to engage with self-service, such as self-collection, to minimise social contact during the delivery process. Thus, we expect that self-collection users who demonstrate the reinforced behavioural pattern will have stronger perceptions of susceptibility and severity. 

Beyond the cognitive assessments, affective components also exist in risk appraisals [29,58]. The affective appraisals address individuals’ emotions associated with health risks, such as fear and anxiety, which deter individuals from performing any risky behaviours. Among these emotions, the anticipatory and anticipated emotions are often distinguished by health researchers [29,59]. *Anticipatory emotions* (e.g., fear and anxiety) are affective reactions to the possibility of harm, whereas *anticipated emotions* (e.g., regret and guilt) are feelings that are expected to occur when risky behaviours are performed [29,60]. Despite the subtle difference, the two types of emotions emerge as distinctive dimensions of affective appraisals in empirical research [29]. In the current study, shoppers may feel anxious if they plan to order home deliveries that require direct interactions with the delivery personnel, which is an example of experiencing a negative anticipatory emotion when appraising the risks of contracting COVID-19. Subsequently, they may regret their decision to receive online orders by means of home deliveries, which is an example of experiencing a negative anticipated emotion. In both scenarios, the affective emotions increase the shoppers’ psychological discomfort, promoting self-collection as a more favourable alternative. Thus, comparing the altered and the maintained self-collection users, we argue that reinforced users experience stronger anticipatory and anticipated emotions. 

Therefore, considering the cognitive and affective components of risk appraisals, we proposed the following hypotheses:

**Hypothesis** **1** **(H1).**  *Susceptibility perception positively contributes to the reinforced behavioural changes in using self-collection services*.

**Hypothesis** **2** **(H2).**  *Severity perception positively contributes to the reinforced behavioural changes in using self-collection services*.

**Hypothesis** **3** **(H3).**  *Anticipatory emotions positively contribute to the reinforced behavioural changes in using self-collection services*.

**Hypothesis** **4** **(H4).**  *Anticipated emotions positively contribute to the reinforced behavioural changes in using self-collection services*.

#### 2.2.2. Planning: Action Planning and Coping Planning

The second stream of health literature highlights the importance of planning in behavioural changes. Planning is defined as a cognitive simulation linking concrete responses to future situations [31]. It involves preparing answers to ‘what-if’ questions. Here, the assumption is that although risk appraisals are effective in creating behavioural motivations, the actual implementation of behavioural changes needs detailed planning [31,32,61]. Such a dual-process (i.e., motivation and implementation) view is reflected in the health action process approach (HAPA). HAPA decomposes the adoption process of health behaviours into two sequential stages: the first stage consists of motivational determinants leading to intention formation, and the second emphasises volitional factors that contribute to health behaviour implementation [62,63]. In fact, an ‘intention–behaviour’ gap is often reported in health literature, and adding planning factors overcomes this gap [31,61,64]. Thus, the insights into risk appraisals and planning factors are complementary in explaining health behaviour adoption. 

Notably, our study does not aim to differentiate the intentional and behavioural outcomes. Instead, we consider the planning as an additional explanatory factor leading to shoppers’ behavioural changes. More specifically, health researchers make a distinction between *action planning and coping planning*: action planning addresses the ‘when’, ‘how’ and ‘how often’ questions in implementing the target behaviour, whereas coping planning concerns how to overcome obstacles in setback situations [31,64]. Moreover, action planning is found to be critical in initiating health behaviours, whereas effective coping planning is necessary for maintaining the behaviours in the long term. Both are important antecedents to foster changes in health behaviours. 

The planning factors are especially relevant in the context of self-service, where no social support is available, and service users are expected to be self-dependent. Applying these two aspects of planning to our study, we determined that effective action planning would familiarise shoppers with the procedure of the self-collection service, such as where to collect and how to collect. Meanwhile, coping planning helps shoppers to be prepared with necessary solutions and response strategies when encountering self-service problems. Therefore, we rationalise that shoppers are more confident in controlling the delivery process through self-collection when they have action and coping plans. The following hypotheses are thus proposed:

**Hypothesis** **5** **(H5).**  *Action planning positively contributes to the reinforced behavioural changes in using self-collection services*.

**Hypothesis** **6** **(H6).**  *Coping planning positively contributes to the reinforced behavioural changes in using self-collection services*.

#### 2.2.3. Subjective Norm

The third stream of the health literature addresses the role of the subjective norm in shaping individuals’ behaviours. Subjective norm refers to the belief that a behaviour is approved by important social others [34]. In different contexts of health research, norm beliefs are associated with behavioural changes [34], and normative interventions are effective in breaking and creating new consumption habits [65]. In fact, the subjective norm is also a critical factor in the theory of planned behaviour, whose validity has been repetitively tested in predicting consumers’ behaviours in general [33,66]. The rationale is that consumers are more likely to behave in line with their social references. In the context of self-collection, shoppers are more likely to use self-collection services when they are perceived to be socially acceptable and favourable. Thus, we argue that the reinforced self-collection users have a stronger perception of subjective norm regarding the use of the service. Thus, the following hypothesis is proposed:

**Hypothesis** **7** **(H7).**  *Subjective norm positively contributes to the reinforced behavioural changes in using self-collection services*.

## 3. Research Method

This study adopts a survey instrument for data collection. A questionnaire is designed and tested by the internal researchers, which is then administered by a professional survey platform online. This section elaborates on the questionnaire design (Section 3.1) and the survey administration process and the generated sample profile (Section 3.2). In Section 3.3, a confirmatory factor analysis is performed to assess the reliability and validity of the measures in the questionnaire. Common method bias is also tested at the end of this section. 

### 3.1. Questionnaire Design 

First, the questionnaire provided an overview about the COVID-19 situation globally and various practices of social distancing locally that impacted shopping activities. At the same time, some illustrations of self-collection lockers were provided using pictures taken from local communities. The purpose was to brief the respondents about our research background and make sure that they had a general idea about the self-collection services even if they were not existing users. 

Next, the survey objective and confidentiality issue were stated, assuring the respondents that all information collected would be anonymised and used solely for the current research. As such, the respondents were encouraged to answer the questionnaire without any self-serving bias. The respondents were then directed to the streaming question that asked about their age; those below 15 years old were automatically rejected, as they were often not financially independent shoppers and were thus disqualified for the current study. 

The qualified respondents would proceed to the main part of the questionnaire. This part consisted of a list of items adapted from the existing literature to measure the proposed affective, cognitive and social factors (see Table 1). More specifically, the risk appraisal factors were measured by nine items adopted from Sheeran, Harris and Epton [29]. It is worth pointing out that Sheeran, Harris and Epton [29] is a meta-analysis based on 208 studies focusing on the four elements of risk appraisal. While it is not the original study of the measures, it is a credible study that identifies and organises the common measures that are shared by multiple studies according to the four elements. For example, fear, worry and anxiety were identified as the key dimensions of anticipatory emotion that were examined by the relevant studies included in the meta-analysis of Sheeran, Harris and Epton [29]. Thus, these items were operationalised as the measurements of anticipatory emotion by incorporating the specific context of delivery service in the current study. Similarly, regret and guilt are used as the measurements of anticipated emotion, physical and psychosocial consequences of contracting COVID-19 as the measurements of severity perception, and general and personal risks of contracting COVID-19 as the measurements of susceptibility perception.

The factors of cognitive planning were measured by the items adopted from Sniehotta, Schwarzer, Scholz and Schüz [31]: three for action planning regarding when, how and how often to use self-collection services; two for coping planning related to potential actions to overcome difficulties in using the service. Finally, the subjective norm was measured by two items concerning personal and injunctive norms [34]. A 7-point Likert Scale was used to capture the respondents’ opinions, ranging from ‘1′ (strong disagreement) to ‘7′ (strong agreement). 

At the end of this part, the respondents were required to indicate their usage frequencies of self-collection services before and during the pandemic with the following options: *never, almost never, yearly, quarterly, monthly, weekly, several times a week and almost every day.* Here, the relatively ambiguous frequency measures were used instead of absolute measures (e.g., times/month) in order to facilitate the participants’ memory recall. These vague measures allowed some extent of uncertainty yet clearly specified several levels of usage intensity, which were especially useful when the participants were unable to recall the exact behavioural frequency. Indeed, respondents are more likely to have a vague memory as to whether they use the self-service on a monthly or on a weekly basis, than to recall the exact times of service usage during a certain period of time. 

It should be highlighted that the survey was conducted in September 2020, which was a period that could be clearly identified as ‘during the pandemic’ by every shopper. This was the period when the pandemic situation had become stabilised and individual shoppers would have adjusted themselves to live with the pandemic in locality where the research was conducted. However, the questionnaire did not specify an exact date as a reference which split the periods of before and during the pandemic. The rationale was that each shopper had his/her own encounter with the pandemic, and shoppers developed their own mental reference as to when the pandemic started to impact on their lives. For example, in Singapore where this study was conducted, there were several rounds of panic buying island-wide from January to March 2020, and the stock-out situations went viral on social media. The government started to enforce strict stay-at-home measures in early April 2020. One might start to feel the influence of the pandemic after knowing the stock-out situations or stay-at-home measure enforcement, and this was his/her personalised split reference of ‘before the pandemic’ and ‘during the pandemic’. It is exactly our intent to capture the impacts of the pandemic which were personally felt by individual shoppers on their self-service usage. In other words, the questionnaire was designed to let each survey respondent use his/her own personalised split reference of the pandemic which was the point when he/she felt the impact of the pandemic. Our research thus focuses on the felt impacts of the pandemic on shoppers’ behaviours. By comparing the respondents’ answers to these two questions, we can categorise the respondents’ behavioural change patterns into the reinforced, the maintained and the altered. Finally, the last part of the questionnaire collected the respondents’ personal information, such as age, gender, household income and education level. 

### 3.2. Survey Administration and Sample Statistics 

The designed questionnaire was internally tested by a group of researchers in related fields. Feedback about the questionnaire’s length, structure and clarity was obtained, and minor adjustments were applied. The final questionnaire was programmed into an online survey, and a professional panel platform was employed for the survey administration. 

Survey invitations were sent to the panellists in Singapore, where the study was conducted. The panellists self-enlisted to participate in the survey by accepting the invitation online. To ensure the data quality, the authors informed the panellists about the survey topic, length and completion reward beforehand. Upon accepting the invitation, the participants would have been aware of the amount of time and effort required for the survey. By doing so, the negative fatigue effect may be minimised. In addition, three attention-checkers were included that asked the participants to select the designated answers (e.g., please select ‘7′ for this question). The purpose was to ensure that participants paid continuous attention to the survey. Those who failed to pass the attention-checkers were automatically terminated, and their data were excluded from further analysis. 

A total of 1143 responses were collected, among which 643 were incomplete due to the respondents being underage, failing attention-check questions or simply losing interest in the middle of the survey. These data were discarded. The remaining 500 valid completes were obtained. A lump sum survey fee was paid to the platform, which included the service charge and respondents’ reward. Notably, a lump-sum payment was made to the survey company for survey programming and data collection. About 30% of the payment was directed to the qualified participants as compensation. Depending on their agreements with the survey platform, the qualified respondents received compensation in cash or other equivalent non-monetary rewards (e.g., mileage). The amount of compensation is comparable to the prevailing market level, and it serves as an appropriate incentive to motivate participation from the targeted sample. 

Table 2 shows the sample statistics. The sample has an equal distribution on gender. About one-third of the participants belong to the 25–34 years old age group, and the sample’s mean age is about 37 years old. Of note, the younger shoppers are more likely to be responsible for the shopping activities during the pandemic period. For example, the adult son/daughter may shop for their parents or other older family members who are more vulnerable to the pandemic. Thus, with an average age of about 37 years old, our sample can be regarded as a good representation of this key shopper group. Moreover, the distribution of different levels of household income is also well-represented by our sample: about 20% of the participants receiving less than S $4000/month, about 20% more than S $12,000, and about 60% in between. 

More importantly, the sample statistics provide some preliminary evidence on shoppers’ changing behaviours towards using self-collection services. Before the pandemic, 33% of participants used the service weekly or daily (i.e., highly frequently users), and this statistic increases to 43% during the pandemic. However, the proportions of non-users and infrequently users remain stable during this period. The statistics seem to suggest that the pandemic has effectively encouraged more frequent usage of self-collection services; however, the segments of non-users and infrequent users are relatively unresponsive to the external stimuli. Further analysis is needed to unveil the changing behavioural patterns. 

### 3.3. Confirmatory Factor Analysis and Common Method Bias Test

Confirmatory factor analysis is conducted to assess the structure of the adapted measurement items. The results are shown in Table 3. The model fit statistics suggest an overall good fit of the measurement model. For example, the absolute fit indices, such as standardised root mean square (SRMR = 0.02) and root mean square error of approximation (RMSEA = 0.05) are below the recommended upper threshold of 0.08; meanwhile, the incremental fit indices, such as comparative fit index (CFI = 0.99) and Tucker-Lewis index (TLI = 0.99), are above 0.95 [67]. 

Next, the reliability of the measures is evaluated by analysing the standardised estimates, composite reliability (CR) and Cronbach’s alpha (CA). As shown in Table 3, the standardised estimates and the values of CRs and CAs are all greater than 0.70, indicating the measures’ adequate level of reliability. 

Furthermore, the measures are also assessed for convergent and discriminant validity. The average variance extracted (AVE) for each construct is calculated. The result shows that all AVEs are above the recommended level of 0.50, thus supporting the convergent validity of the measures. Table 4 shows the square roots of AVEs and the construct correlations along and below the main diagonal, respectively. The square roots of AVEs are larger than the construct correlations; hence, the discriminant validity is also confirmed [67]. 

In addition, as the survey was used for data collection, common method bias needs to be tested. In this regard, we adopt Harman’s single factor method, where a confirmatory factor analysis is performed again based on a single factor model. The single-factor model produced the following fit indices: χ2 = 4249.30, df =324, χ2/df = 13.12, CFI = 0.53, IFI = 0.53, SRMR = 0.15, RMSEA = 0.16. The results show that this model has a considerably worse fit with the data. Thus, the common method bias is unlikely to be a major issue for our study. 

## 4. Findings and Discussion

This study employs a combined method to analyse and visualise the research findings, including ANCOVA test, multinomial logistic regression and radar chart. More specifically, in Section 4.1, an ANCOVA test is firstly performed to validate the differentiated impacts of affective, cognitive and social factors on the behavioural change patterns of self-collection users while controlling some unhypothesised effects. The results are then visualised using radar charts. In Section 4.2, we conduct a multinomial logistic regression to profile the self-collection users based on their behavioural changes (i.e., reinforced, maintained and altered) and to determine the explanatory powers of the proposed contributing factors. Finally, Section 4.3 presents the results of some post-hoc analyses based on ANOVA. The analysis results further profile the maintained users focussing on their self-collection frequencies (i.e., frequent users vs. infrequent users).

### 4.1. Initial Validation and Descriptive Statistics 

By comparing usage frequencies of self-collection service before and during the pandemic, we identified the following three behavioural change patterns of the self-collection users: the reinforced (i.e., frequency increased), the maintained (i.e., frequency unchanged), and the altered (i.e., frequency dropped). 

Among the 500 sampled users, 151 users indicated that they used the service more frequently than before; more than half of the sample maintained the usage frequency of self-collection service during the pandemic (*n* = 293); and a small proportion of the respondents showed a declining interest in using the service (*n* = 53). Thus, the result suggests that the pandemic does promote the usage of self-collection service to a considerable extent; however, the majority of users’ behavioural pattern remains unchanged. 

An ANCOVA test is then applied to determine the statistical differences regarding the perceptions on the affective, cognitive and social factors proposed to influence the self-collection users’ behavioural patterns. The method of ANCOVA is considered an extension of ANOVA by including additional covariates as the control variables in the statistical model. In the current study, shoppers’ usage of self-collection service may be influenced by their online shopping frequency, that is, more (less) frequent online shopping may result in reinforced (altered) usage of self-collection service. Herein, it is important to control the effect related to changes in online shopping frequency. Thus, shoppers’ behavioural change of online shopping before and during the pandemic was held as the control variable in the ANCOVA test. 

The ANCOVA test results in Table 5 reveal that all proposed factors convey different meanings statistically (*p* < 0.05) to the three user groups. In fact, reinforced users rate all the factors higher than the respective mean scores, suggesting that they were under heightened risk appraisals (i.e., perceived anticipatory/anticipated emotions, and perceived severity/susceptibility), they were better prepared in both action and coping planning, and they perceived the social norm of self-service as more prevalent. In addition, it is found that online shopping frequency change, as the control variable, does lead to behavioural changes of self-collection service when the factors of perceived severity, anticipated emotion and planning are concerned. Yet, upon controlling the impacts of online shopping frequency change, our proposed factors remain significant predictors for shoppers’ behavioural changes of self-collection service. Therefore, the ANCOVA test provides the initial evidence that all proposed factors contribute to the behavioural changes of self-collection users. 

**Figure 1:**
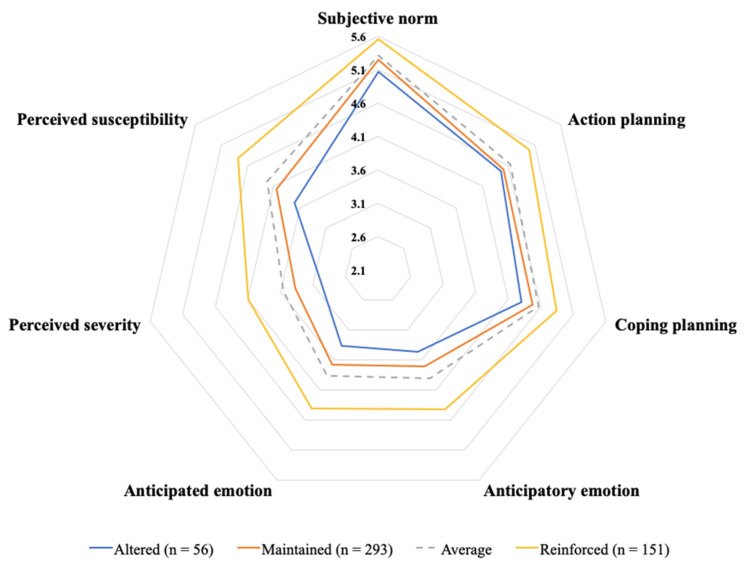
Descriptive statistics (altered, maintained and reinforced).

To better visualise the perceptual differences, we produced a radar chart by plotting the perception scores of the proposed factors by the three user groups. A dotted grey circle represents the mean scores as a reference. For easy interpretation, the total area of the circle may be viewed as the aggregated influence of the contributing factors perceived by the self-collection users. As shown in Figure 1, the perception scores of the seven factors by the reinforced users (i.e., yellow circle) jointly form the outer circle with the largest area. The two inner circles (i.e., blue and orange circles) reflect the perceptions by the altered and maintained users, with the altered users forming the smallest circle. Herein, the radar chart clearly demonstrates the perceptual differences among the reinforced, maintained and altered self-collection users. In other words, the reinforced users receive the strongest aggregated influence of the affective, cognitive and social factors, followed by the maintained users and the altered users. Thus, in line with the ANCOVA test, the radar chart also confirms the impacts of the seven factors on the changes of usage patterns of self-collection services. 

### 4.2. Multinomial Logistics Regression 

Furthermore, we employ multinomial logistics regression to test the hypotheses and assess the proposed factors’ explanatory power in predicting users’ behavioural changes. The three behavioural change patterns, that is, the reinforced (baseline), the altered and the maintained, are held as the dependent variable. The seven proposed factors (i.e., four factors related to affective/cognitive appraisals each, two factors related to cognitive planning, and one factor of subjective norm) are held as the independent variables. Of note, seven separate regression models are constructed by including one independent variable in each model. The purpose is to evaluate the differentiated impact of each factor on behavioural changes of self-collection users. The analysis results are shown in Table 6. 

Referring to the model fitting information, all seven models demonstrate a good fit with the data based on the chi squared test with two degrees of freedom. The results indicate that each model does a good job of explaining self-collection users’ behavioural changes. Thus, the proposed hypotheses are accepted. However, the values of Pseudo-R^2^ (Nagelkerke) are relatively low (varying from 2% to 6%), suggesting that each proposed factor accounts for a small proportion of the variance in users’ behavioural changes. This is in line with the health literature [29], which acknowledges the relatively weak explanatory power of health concerns in behavioural changes. In addition, since self-collection is essentially a commercial service, it is understandable that stronger service-related factors may exist which dominate shoppers’ decision-making process. Additionally, the data used in this study were collected in Singapore, which is not the epicentre of the pandemic. The impact of the pandemic on shoppers’ behaviours may be limited, and the pandemic-driven behavioural changes may be less prominent. This also explains the relatively weak explanatory power of the proposed predictors. Nonetheless, the proposed factors in the current study represent a new source of behavioural change that provides additional explanatory power on top of service-dominated considerations. Thus, the salience of these factors in predicting pandemic-induced self-service behaviours should not be ignored. 

In terms of the explanatory power based on Pseudo-R^2^, risk appraisals seem to be the strongest predictors: anticipatory and anticipated emotions explain 4% of the variance, and severity and susceptibility perceptions account for 6%. The planning and social factors turn out to be of weaker predictive power, with values of Pseudo-R^2^ of about 3% or less. Therefore, we may infer from the results that risk appraisals, particularly cognitive components, are more effective in motivating consumers’ engagement with self-service. 

Furthermore, the values of Exp(B) are analysed. Exp(B) reflects the odds ratio (OR) as to whether a particular behavioural change pattern is more (when OR greater than 1) or less (when OR less than 1) likely to have a certain characteristic than the baseline alternative. For example, the OR for the subjective norm in the altered user group is 0.69 (*p* < 0.01). With one unit increment of perceived subjective norm, the value suggests that the users are less likely to belong to the altered group (69%) than the baseline group of the reinforced (default likelihood of 100%). In other words, the higher the perceived subjective norm, the less (more) likely for the users to decrease (increase) the usage of self-collection services. Referring to the Exp(B) column in Table 6, when holding *the reinforced* as the baseline, we determine that the Exp(B) values of all factors are less than 1. To interpret, users are likely to increase their usage of self-collection services with heightened affective/cognitive risk appraisals, more effective action/coping planning and stronger perceived subjective norm. Thus, as we hypothesised that the affective, cognitive and social considerations collectively promote shoppers’ engagement behaviours with self-collection. 

### 4.3. Post Hoc ANOVA Test 

Taking a closer look into the maintained groups, we observe heterogeneities in behavioural change patterns even within the same group. The maintained users may be further subcategorised based on their usage frequencies of self-collection services. More specifically, shoppers who used the service weekly or more are labelled as frequent users (*n* = 98). Meanwhile, those who used the service less frequently (i.e., on a monthly basis or less) are labelled as infrequent users (*n* = 113). In addition, a subsample of 82 shoppers who remained non-users during the pandemic is also identified. Referring to Table 7, frequent users seem to rate all seven factors that are higher than the infrequent users, with the non-users giving the lowest ratings. The ANOVA result confirms the significant differences in the ratings given by the three subsamples to the seven factors. Besides, the results shown in Table 7 point to an unexpected yet interesting observation that the frequent users of self-collection services seem to be naturally more health-conscious than the infrequent or non-users. This might be due to a relatively higher level of personal hygiene requirement by frequent self-collection users. As a result, they favour self-service over any interactions with service personnel. Also, as frequent users of a rather new delivery alternative based on self-collection, these shoppers are more likely to be open to new information and have better exposure to the COVID-related information during the pandemic. Thus, they may have more concerns about the health risks of COVID-19.

**Figure 2:**
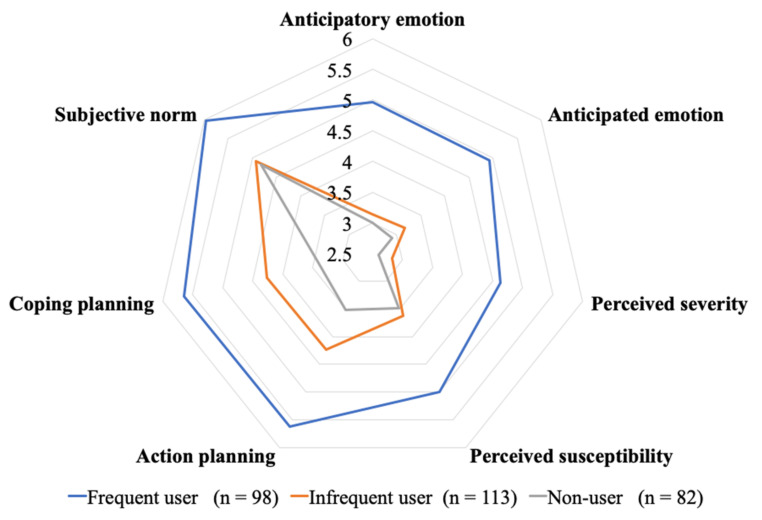
Descriptive statistics (frequent, infrequent, and non-users).

The radar chart (see Figure 2) reveals a similar pattern where the perception scores of the frequent users form the outer circle (in blue), and the infrequent users’ and non-users’ scores form the two inner circles. To this end, we may suggest that the proposed affective, cognitive and social factors also contribute to self-collection usage frequencies as maintained by shoppers.

## 5. Conclusions

This study explores the pandemic-driven changes in shoppers’ usage of self-service. The research context of e-commerce self-collection was carefully selected to emphasise shoppers’ self-service commitment while deemphasising the technological hurdle. We consult the health literature to explain shoppers’ behavioural change patterns in using self-collection services during the pandemic. In particular, theoretical insights from the studies of risk appraisals, planning and subjective norm are integrated, forming an affective–cognitive–social perspective of pandemic-driven changes. To answer the research question ‘Does COVID-19 promote a self-service spirit among modern shoppers?’, our findings are summarised as follows:

- Based on analysis in Section 4.1, a considerable proportion of shoppers (151 out of 500) increased their usage frequencies of self-collection services, whereas the usage frequencies by the majority shoppers (293 out of 500) remained unchanged.
- Based on analysis in Section 4.2, as hypothesised, the reinforced users are associated with higher risk appraisals (susceptibility/severity perceptions and anticipatory/anticipated emotions), action/coping planning, and perceived subjective norm. Meanwhile, all the proposed factors demonstrate a relatively weak explanatory power (2–6%).
- Based on the analysis in Section 4.3, the proposed factors also contribute to the maintained usage frequencies of self-collection services; that is, the users who maintained at high usage frequencies (weekly or more) tended to have a higher level of affective/cognitive risk appraisals, a better action/coping planning and a stronger subjective norm perception.

### 5.1. Theoretical Contribution

By integrating health concerns into the service literature, this study recognises the compatibility of self-service with the pandemic situation and emphasises shoppers’ health concerns as the salient motivators for behavioural changes. Our study suggests that shoppers’ engagement with self-service shares great similarities with their adoption of health behaviours, where risk appraisals, planning and normative beliefs are crucial. Indeed, the pandemic has heightened consumers’ health consciousness, which alleviates the importance of health considerations in almost every consumption decision. In this regard, we extend the self-service studies by integrating the relevant health theories. At the same time, we argue for increasing relevancy of health literature to the mainstream consumer studies, which have been dominated by service and marketing literature. 

By validating an affective-cognitive-social perspective of pandemic-driven behavioural changes, we contribute to the self-service literature with an affective-cognitive-social perspective, which is especially relevant in the context of COVID-19. To elaborate, our research concurs with the risk appraisal studies that both affective responses and cognitive assessments cause shoppers’ behavioural changes [29]. This finding is in line with the self-collection literature, where the dual elements (i.e., affect and cognition) elicit shoppers’ voluntary participation in self-collection [25]. It also echoes the self-service literature in general, where utilitarian and emotional considerations are emphasised [11,68]. Additionally, our study further validates a planning perspective, which has been a recurring theme in the health literature [31,64,69], but has seldom been considered in self-collection studies or in the self-service literature in general. Shoppers’ action and coping planning in using the self-collection services is suggested to complement their risk appraisals leading to the implementation of behavioural changes. Finally, being a public health crisis, the pandemic prompts the normative beliefs from a background presence to a forefront consideration in shoppers’ behaviours. Self-service has become more than a private service option, being a socially desirable and responsible behaviour. In this connection, this study confirms the salient influence of the subjective norms in shoppers’ usage of self-collection services.

### 5.2. Managerial Implications 

Practically, our findings provide managerial implications for self-collection service operators and retailers. Although the pandemic causes major service disruptions, it also creates rare opportunities to foster desirable behavioural changes among shoppers. 

For self-collection operators, this opportunity should be taken to promote the service, emphasising the service’s benefits, such as its contactless nature and flexibility by way of self-service. As revealed by the sample statistics, when comparing the usage frequency of self-collection services before and during the pandemic, the frequent users seem to be responsive to the pandemic by increasing their usage frequencies, whereas the non-users and the infrequent users are less responsive. To this end, additional stimuli may be needed to motivate the less responsive user groups. Planning factors may be emphasised in this regard. For example, the operators may collaborate with the e-commerce website to advertise the self-collection services, focussing on the collection procedures and available backend support during collection. They can specifically target non-users who may be unfamiliar with the service. By doing so, the non-users may have a better idea about the service, helping them plan to use the service accordingly. 

For the retailers, our study suggests that about 30% of the surveyed shoppers increase their usage of self-service during the pandemic. As such, retailers should accommodate the shoppers’ changing preferences by providing more self-service tools and options. As health considerations (e.g., susceptibility and severity perceptions) partly drive preferences, retailers should ensure a high level of hygiene of the self-service facilities by applying regular disinfection, and informing users accordingly. However, our study also reveals that some shoppers have remained non-users of self-service (82/500) during the pandemic. Their preference for conventional services should also be respected. Thus, retailers should plan to entertain shoppers with diverging preferences regarding self-service and strategically allocate resources to self-service and service staff. 

### 5.3. Limitations 

This research has several limitations. Firstly, an online survey is used in this study. As a result, the older population, who are less familiar with online tools, are underrepresented in our sample. While an online survey seems to be the most feasible way to gather opinions from a large sample group given the ongoing pandemic situation, the limitation concerning the representativeness of the sampled population should be highlighted. Thus, our findings may be more applicable to the younger shopper group, whereas further research may be needed using a boosted sample from the older shoppers to extend the findings to the general population. 

Secondly, this study collects information of shoppers’ self-service usage before the pandemic based on their memory recalls. While vague measures were adopted to facilitate the recall process, the level of accuracy in the participants’ answers may vary. The embedded limitation associated with the recall-based research design should be noted. 

Thirdly, the factors proposed in this study serve as significant yet relatively weak predictors to shoppers’ behavioural change. This is in line with the health literature [29], which acknowledges the weak explanatory power of health concerns in behavioural changes. Future research may extend our study by incorporating more insights from health studies to predict shoppers’ behaviours. The interactions among the predictors may also be explored. Furthermore, although our study confirms the impact of the pandemic on shoppers’ behavioural changes, it remains to be explored as to whether such changes are transitory beyond the pandemic period. As raised by Sheth [1], the critical question ‘will the old habits return or die?’ is worth continuous attention from future researchers. 

In addition, the newly formed category of behavioural change is not included in the current study given the maturity level of self-collection services in the research context. However, it could be a behavioural change pattern of key interest in the emerging self-collection market. It is encouraged that future researchers take the newly formed user group into consideration especially when establishing self-collection services where missing. 

Finally, the study selectively uses self-collection services as the representative self-service, given the high level of self-service commitment required. Yet, the specific characteristics of e-commerce delivery may restrict the generalisability of the self-collection services to the wider context of self-service in general. Thus, the research context should be kept in mind when interpreting/generalising the findings. 

## Figures and Tables

**Table 1:** Measures and descriptions.

Construct	Measure	Description	Source
Perceived susceptibility	SUS-GEN	People tend to contract the COVID-19 virus when they interact face-to-face with others frequently	Sheeran, Harris and Epton [29]
	SUS-PER	My chances of contracting the COVID-19 virus are high when I interact face-to-face with others frequently	
Perceived severity	SEV-PHY	My physical health would be negatively influenced if I contracted the COVID-19 virus	
	SEV-SOC	My social relationships would be negatively influenced if I contracted the COVID-19 virus	
Anticipatory emotion	EMO-FEA	For my next purchase, I would fear to interact face-to-face with delivery personnel due to COVID-19	
	EMO-WOR	For my next purchase, I would feel worried to interact face-to-face with other shoppers due to COVID-19	
	EMO-ANX	For my next purchase, I would feel anxious to shop in physical settings due to COVID-19	
Anticipated emotion	EMO-REG	I would feel regretful if I ordered home delivery when self-collection services are available	
	EMO-GUI	I would feel guilty if I shopped in physical settings when self-collection services are available	
Action planning	PLA-ACT1	I have made a detailed plan regarding when to use the self-collection services	Sniehotta, Schwarzer, Scholz and Schüz [31]
	PLA-ACT2	I have made a detailed plan regarding how to use the self-collection services	
	PLA-ACT3	I have made a detailed plan regarding how often to use the self-collection services	
Coping planning	PLA-COP1	I have made a detailed plan regarding how to cope with possible setbacks when using the self-collection services	
	PLA-COP2	I have made a detailed plan regarding what to do in difficult situations to act according to my intentions	
Subjective norm	NOR-PER	I strongly feel that I should use the self-collection services	Mosler [34]
	NOR-INJ	Overall, I think people who are important to me would encourage me to use the self-collection services	
Usage frequency	Before and Now	How often did (do) you use self-collection service before (during) the pandemic? Never; Almost never; On a yearly basis; On a quarterly basis; On a monthly basis; On a weekly basis; Several times a week; Almost every day	Designed by this study

**Table 2:** Sample statistics.

Sample Characteristics	Frequency	Percentage (%)
**Gender**		
Male	250	50
Female	250	50
**Age (years)**		
16–24	92	18
25–34	183	37
35–44	122	24
45–54	73	15
>55	30	6
*Average age*	37.12	
**Household income (SGD/month)**		
<3999	93	19
4000–7999	156	31
8000–11,999	139	28
12,000–19,999	74	15
>20,000	38	8
**Frequency of using self-collection service (before)**		
Non-user	92	18
Infrequent users (almost never to yearly)	100	20
Moderately frequent users (quarterly to monthly)	144	29
Highly frequent users (weekly to daily)	164	33
**Frequency of using self-collection service (now)**		
Non-user	92	18
Infrequent users (almost never to yearly)	84	17
Moderately frequent users (quarterly to monthly)	108	22
Highly frequent users (weekly to daily)	216	43

**Table 3:** Confirmatory factor analysis.

Construct	Measure	Standardised Estimate	*t*-Value	AVE	CR	CA
Perceived susceptibility	SUS-PER	0.89	-	0.80	0.89	0.89
	SUS-GEN	0.90	27.59			
Perceived severity	SEV-SOC	0.91	-	0.86	0.92	0.92
	SEV-PHY	0.94	35.15			
Anticipatory emotion	EMO-FEA	0.97	43.86	0.91	0.97	0.97
	EMO-WOR	0.97	44.13			
	EMO-ANX	0.92	-			
Anticipated emotion	EMO-REG	0.92	41.89	0.89	0.94	0.95
	EMO-GUI	0.97	-			
Action planning	PLA-ACT1	0.96	44.70	0.9	0.97	0.97
	PLA-ACT2	0.96	44.94			
	PLA-ACT3	0.93	-			
Coping planning	PLA-COP1	0.96	48.57	0.92	0.96	0.96
	PLA-COP2	0.96	-			
Subjective norm	NOR-PER	0.74	14.25	0.67	0.80	0.79
	NOR-INJ	0.89	-			

Model fit statistics: χ^2^ = 173.53, df = 83, χ^2^/df = 2.09, GFI = 0.96, CFI = 0.99, IFI = 0.99, TLI = 0.99, SRMR = 0.02, RMSEA = 0.05, AVE, average variance extracted; CR, composite reliability; CA, Cronbach’s alpha.

**Table 4:** Average variance extracted and construct correlation.

Construct	Anticipatory Emotion (1)	Anticipated Emotion (2)	Perceived Severity (3)	Perceived Susceptibility (4)	Action Planning (5)	Coping Planning (6)	Subjective Norm (7)
1	0.95 ^a^						
2	0.88 ^b^	0.94					
3	0.85	0.88	0.93				
4	0.83	0.80	0.84	0.89			
5	0.53	0.55	0.50	0.43	0.95		
6	0.54	0.55	0.52	0.45	0.90	0.96	
7	0.55	0.54	0.48	0.50	0.60	0.57	0.82

^a^ Square roots of AVEs are shown along the diagonal ^b^ Construct correlations are shown below the diagonal.

**Table 5:** ANCOVA test results (altered, maintained and reinforced users).

Factor	Reinforced (*n* = 151)	Maintained (*n* = 293)	Altered (*n* = 56)	Mean	F-Score	*p*-Value	*p*-Value of Control Variable ^
Perceived susceptibility	4.78	4.04	3.70	4.23	8.74	***	n.s.
Perceived severity	4.10	3.37	2.97	3.55	9.65	***	*
Anticipatory emotion	4.42	3.71	3.47	3.90	7.34	***	n.s.
Anticipated emotion	4.40	3.68	3.36	3.87	8.83	***	*
Action planning	4.99	4.50	4.46	4.64	6.37	***	**
Coping planning	4.85	4.48	4.30	4.57	6.85	***	***
Subjective norm	5.55	5.24	5.07	5.31	3.60	*	n.s.

* *p* < 0.05, ** *p* < 0.01, *** *p* < 0.001, n.s. *p* > 0.05, ^ Change in online shopping frequency as the control variable.

**Table 6:** Multinomial logistic regression analysis.

		B	*p*-Value (Factor)	Exp(B)	Model Fitting Information		Hypothesis Test Result		Pseudo R-Square
Construct					Chi-Square (df = 2)	Chi-Square (df = 2)	Hypothesis	Test Result	
Perceived susceptibility	Altered	−0.39	***	0.68	25.38	***	H1	Supported	6%
	Maintained	−0.27	***	0.77					
Perceived severity	Altered	−0.37	***	0.69	23.53	***	H2	Supported	6%
	Maintained	−0.23	***	0.79					
Anticipatory emotion	Altered	−0.28	**	0.75	18.17	***	H3	Supported	4%
	Maintained	−0.21	***	0.81					
Anticipated emotion	Altered	−0.32	***	0.73	20.07	***	H4	Supported	4%
	Maintained	−0.21	***	0.81					
Action planning	Altered	−0.23	*	0.8	11.03	**	H5	Supported	3%
	Maintained	−0.21	**	0.81					
Coping planning	Altered	−0.23	*	0.8	7.45	*	H6	Supported	2%
	Maintained	−0.16	*	0.85					
Subjective norm	Altered	−0.37	**	0.69	10.56	**	H7	Supported	3%
	Maintained	−0.25	**	0.78					

* *p* < 0.05, ** *p* < 0.01, *** *p* < 0.001.

**Table 7:** Heterogeneity in the maintained users (ANOVA result).

Construct	Maintained (*n* = 293)				
	Frequent User (*n* = 98)	Infrequent User (*n* = 113)	Non-User (*n* = 82)	F-Score	*p*-Value
Anticipatory emotion	4.96	3.14	3.00	17.81	***
Anticipated emotion	4.93	3.16	2.91	21.88	***
Perceived severity	4.63	2.83	2.60	22.98	***
Perceived susceptibility	4.99	3.63	3.48	13.46	***
Action planning	5.62	4.23	3.52	44.33	***
Coping planning	5.65	4.26	3.38	58.36	***
Subjective norm	5.96	4.92	4.82	15.95	***

Frequent User: Use on a weekly basis or more; Infrequent User: Use on a monthly basis or less. *** *p* < 0.001.

## Data Availability

Data shall be made available upon request from the lead author.
